# Supplementary material for: Harnessing Nanomedicine to Potentiate the Chemo-Immunotherapeutic Effects of Doxorubicin and Alendronate Co-Encapsulated in Pegylated Liposomes
Source: Pharmaceutics. 2023 Nov 9;15(11):2606. doi: 10.3390/pharmaceutics15112606 (PMC10675201; doi:10.3390/pharmaceutics15112606)

Supplement Figures

Gabizon et al.

Figure S1:
In vitro growth curve of Wehi-164 mouse sarcoma cells exposed for 72 h to free Dox, PLD or PLAD

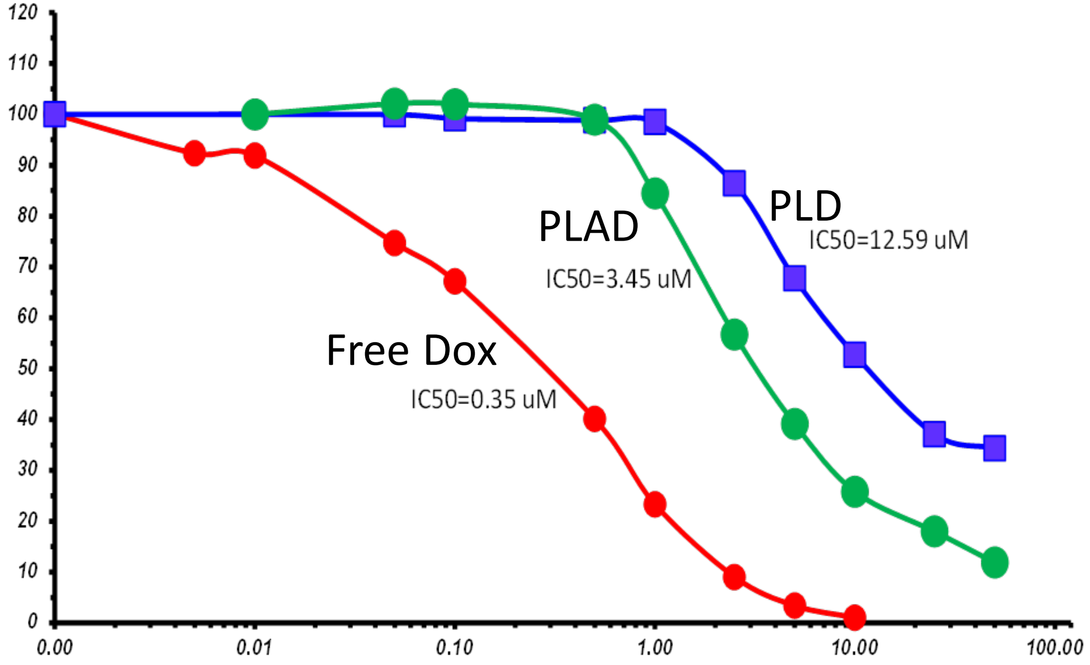

Figure S2:
Antitumor activity of PLAD combined with Vγ9Vδ2 T cells (for further rdetails, see Figure 9A-B)

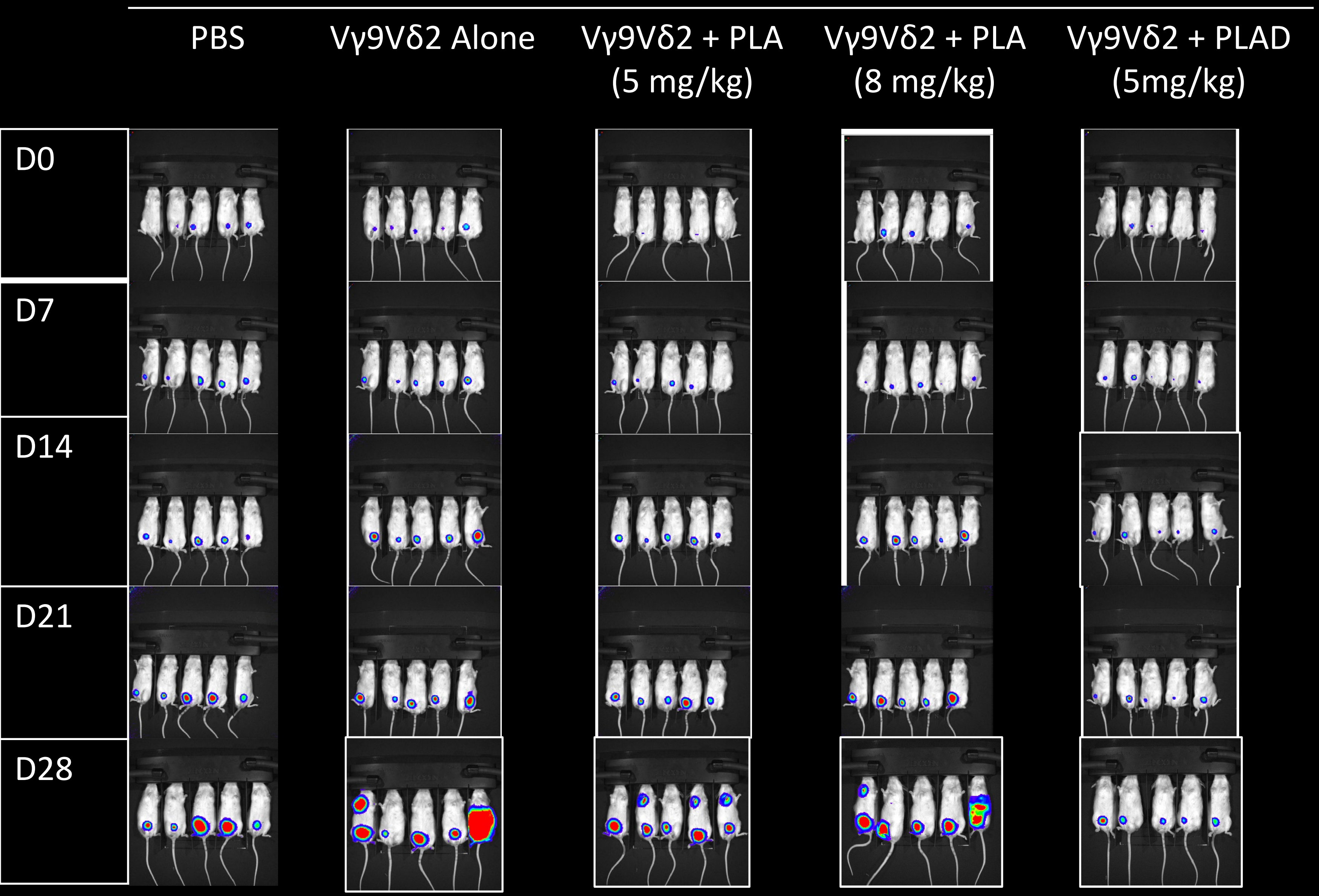

Supplement: Supplementary file 1 [file pharmaceutics-15-02606-s001.zip › pharmaceutics-2673276-supplementary.pdf]
